# A Comparative Evaluation of the Cyclic Fatigue Resistance of Three Nickel-Titanium Rotary File Systems in a Simulated Curved Canal: An In Vitro Study

**DOI:** 10.7759/cureus.113866

**Published:** 2026-08-03

**Authors:** Ieeshan Farooq Shah, Panna Mangat, Faizan Masarat, Fariha Fatima, Viswesh Subramani, Jasleen Kaur

**Affiliations:** 1 Department of Conservative Dentistry and Endodontics, Kalka Dental College, Meerut, IND; 2 Department of Orthodontics and Dentofacial Orthopedics, Titoriya Dental Clinic, Meerut, IND

**Keywords:** endodontics, fatigue, materials testing, nickel-titanium, root canal preparation

## Abstract

Introduction: Nickel-titanium (NiTi) rotary instruments have significantly improved the efficiency and predictability of root canal preparation because of their superior flexibility and shape-memory characteristics. Despite these advantages, unexpected instrument separation caused by cyclic fatigue remains a major clinical concern, particularly during instrumentation of curved root canals. Continuous advancements in heat treatment and instrument design have led to the introduction of newer rotary systems with improved mechanical properties. However, comparative evidence regarding their cyclic fatigue resistance remains limited. This study aimed to compare the cyclic fatigue resistance of three NiTi rotary file systems in a standardized simulated curved canal under in vitro conditions.

Methods: An in vitro study was conducted using 60 new NiTi rotary files equally allocated into three groups (n = 20): GenEndo, Orodeka Plex, and ProTaper Gold. All instruments were tested in a custom-fabricated stainless steel artificial canal with a standardized curvature under continuous rotation using manufacturer-recommended speed and torque settings. The time to fracture was recorded using a digital stopwatch, and the number of cycles to fracture (NCF) was calculated. Representative fractured specimens were qualitatively examined using scanning electron microscopy (SEM). Data were analyzed using one-way analysis of variance (ANOVA) followed by Tukey's post hoc test, with statistical significance set at p < 0.05.

Results: ProTaper Gold demonstrated the longest mean time to fracture (806.80 ± 58.65 seconds), followed by Orodeka Plex (514.65 ± 51.56 seconds) and GenEndo (406.80 ± 66.02 seconds). Orodeka Plex exhibited the highest mean NCF (4153.25 ± 421.74), followed by ProTaper Gold (3962.40 ± 297.80) and GenEndo (2619.20 ± 437.56). One-way ANOVA revealed statistically significant differences among the three rotary systems for both the time to fracture and NCF (p < 0.001). Post hoc analysis demonstrated significant pairwise differences in time to fracture among all groups, whereas Orodeka Plex and ProTaper Gold showed comparable NCF values (p = 0.278). SEM examination revealed characteristic cyclic fatigue fracture features, including crack initiation zones, fatigue striations, and overload regions, in all tested instruments.

Conclusion: The tested rotary file systems exhibited significantly different cyclic fatigue behaviors under standardized laboratory conditions. ProTaper Gold demonstrated the greatest resistance in terms of fracture time, whereas Orodeka Plex achieved the highest NCF. GenEndo showed a comparatively lower fatigue resistance. Instrument metallurgy, heat treatment, rotational speed, and geometric design appear to play important roles in determining the fatigue performance of NiTi rotary instruments.

## Introduction

Nickel-titanium (NiTi) rotary instruments have revolutionized root canal preparation by providing superior flexibility, shape memory, and cutting efficiency compared with conventional stainless-steel hand instruments [1]. Their ability to negotiate curved root canals while preserving the original canal anatomy has significantly improved the quality and predictability of endodontic treatments. Despite these advantages, unexpected instrument fracture remains one of the most challenging procedural complications during root canal therapy, as it may hinder the effective cleaning and shaping of the canal system and negatively influence treatment outcomes [2].

Fracture of NiTi rotary instruments generally occurs owing to torsional stress or cyclic fatigue. Cyclic fatigue develops when an instrument repeatedly undergoes alternating tensile and compressive stresses while rotating within a curved canal [3]. Over time, these repetitive stresses initiated microcracks that propagated until catastrophic fractures occurred without visible signs of deformation. Consequently, cyclic fatigue resistance has become one of the most important mechanical properties for evaluating the clinical safety and durability of NiTi rotary instruments [4].

Recent advances in metallurgy, manufacturing processes, cross-sectional design, and thermal treatment technologies have led to the development of new generations of rotary instruments with improved mechanical performance. Heat-treated alloys, modified geometric designs, and optimized manufacturing techniques have been introduced to enhance flexibility and fatigue resistance while maintaining cutting efficiency [5,6]. However, differences in alloy composition, taper, cross-sectional configuration, and proprietary heat-treatment protocols may result in considerable variations in the fatigue behavior of different file systems [6]. Therefore, independent laboratory evaluations are essential to provide clinicians with evidence-based information regarding mechanical performance.

Static cyclic fatigue testing using standardized artificial canals is widely accepted as a reliable method for comparing the fatigue resistance of rotary instruments under controlled experimental conditions [7]. Such testing minimizes operator-related variability and allows for direct comparison among different instrument systems. Although numerous studies have investigated commercially available NiTi rotary files, evidence regarding the comparative fatigue resistance of recently introduced systems remains limited [3,4,7]. Evaluation of these instruments under standardized conditions may assist clinicians in selecting safer and more durable rotary files for routine endodontic practice.

The aim of the present in vitro study was to compare the cyclic fatigue resistance of three NiTi rotary file systems in a standardized simulated curved canal. Specifically, this study sought to determine and compare the time to fracture and the number of cycles to fracture (NCF) of the tested rotary instruments under identical experimental conditions. Furthermore, we aimed to evaluate whether statistically significant differences existed in cyclic fatigue resistance among the three file systems and to identify the instrument demonstrating the greatest resistance to cyclic fatigue, thereby providing evidence to support the selection of safer and more durable rotary instruments for clinical endodontic practice.

## Materials and methods

The present experimental study was designed as an in vitro process conducted in the Department of Conservative Dentistry and Endodontics, Kalka Dental College, Meerut, India, between May 2024 and February 2025. As the study did not involve any human or human tissue, ethical approval was not required for this study. The sample size was estimated a priori using G*Power software version 3.1 (Heinrich Heine University Düsseldorf, Düsseldorf, Germany) based on the F-test family for one-way analysis of variance (ANOVA) with fixed effects. The calculation was performed using an effect size (f) of 0.30 derived from previously published literature evaluating the cyclic fatigue resistance of NiTi rotary instruments, an alpha error probability of 0.05, a statistical power (1-β) of 80%, and three comparison groups [8]. The minimum required sample size was calculated as 60 rotary instruments, with 20 instruments allocated to each study group.

Three commercially available heat-treated NiTi rotary file systems with comparable apical size and taper characteristics were included in the study. Group I consisted of GenEndo files (size 25, 0.06 taper, 25 mm length; Micro-Mega, France), Group II comprised Orodeka Plex files (size 25, 0.06 taper, 25 mm length; Orodeka, Jining, China), and Group III included ProTaper Gold F2 files (tip size 25, variable progressive taper with an apical taper of 0.08, 25 mm length; Dentsply Sirona, Tulsa, OK). All instruments were sterile, unused, and obtained directly from authorized distributors to ensure authenticity and batch uniformity. Before testing, each file was examined under 20× magnification using a stereomicroscope (Olympus Corporation, Tokyo, Japan) to identify manufacturing defects, unwinding, distortion, surface irregularities, and other visible imperfections. Instruments exhibiting structural defects were excluded and replaced with new instruments from the same manufacturing batch to maintain consistency throughout the experiment.

Only new unused NiTi rotary files with intact cutting blades, uniform geometry, and no visible manufacturing defects were included in the study. Instruments that exhibited deformation, unwinding, corrosion, scratches, defects in cutting flutes, damaged tips, or any evidence of previous handling or contamination were excluded. Furthermore, instruments from different manufacturing batches were not included to eliminate the variability arising from batch-to-batch differences in metallurgical properties.

Cyclic fatigue testing was performed using a custom-fabricated stainless steel artificial canal specifically designed to simulate a severely curved root canal under standardized conditions. The test block was manufactured from hardened stainless steel to ensure dimensional stability throughout the experimental procedure. The artificial canal possessed a fixed angle of curvature of 60° with a 5-mm radius of curvature, which is widely employed in cyclic fatigue research to reproduce clinically relevant stress concentrations encountered during instrumentation of curved canals. The internal diameter of the canal was slightly larger than that of the tested instruments to permit unrestricted rotation while ensuring intimate contact between the instrument and canal wall at the point of maximum curvature. The center of curvature, canal depth, and working length were standardized for all specimens, so that each instrument was subjected to identical mechanical loading conditions. The canal was covered with a transparent tempered glass plate during testing to prevent instrument displacement, while simultaneously allowing uninterrupted visualization of file rotation and fracture.

Prior to testing, a synthetic high-flow lubricant (WD-40 Specialist Silicone Lubricant, WD-40 Company, San Diego, CA) was uniformly applied within the artificial canal to minimize frictional heat generation and reduce metal-to-metal abrasion between the instrument and stainless-steel canal wall. The lubricant was reapplied before each instrument was tested to maintain identical testing conditions throughout the experiment. Each rotary file was mounted on a torque-controlled endodontic motor (X-Smart Plus Endodontic Motor, Dentsply Sirona Endodontics, Ballaigues, Switzerland) operated according to the manufacturer's recommended rotational speed and torque settings. ProTaper Gold files were rotated at 300 rpm with a torque of 2.0 N.cm, GenEndo rotary files were operated at 400 rpm with a torque of 2.5 N.cm, and Orodeka Plex files were rotated at 500 rpm with a torque of 2.5 N.cm. Each instrument was introduced into the artificial canal until the tip reached the predetermined working length and was allowed to rotate continuously under static conditions without any pecking or axial movement until fracture occurred. All the tests were conducted at room temperature under identical environmental conditions by the same experienced operator to minimize operator-dependent variability.

The time required for fracture of each instrument was measured in seconds using a calibrated digital stopwatch (Casio Computer Co. Ltd., Tokyo, Japan). To eliminate observational bias, a second independent investigator simultaneously observed the rotating instrument and confirmed the exact moment of instrument separation. The NCF, which represented the primary outcome variable, was calculated using the following formula:

$\mathrm{NCF} = \frac{\text{Rotational speed (rpm)} {\times} \text{Time to fracture (second)}}{60}$

The time to fracture and the calculated NCF were recorded for every specimen on a standardized data collection sheet immediately after the completion of each test. Following the fracture, the fractured fragments were carefully retrieved from the artificial canal using nonserrated tweezers to prevent surface damage. The fractured segments were ultrasonically cleaned in absolute ethanol for five minutes to remove lubricant residues and debris, followed by air-drying at room temperature [9]. Representative fractured specimens from each study group were mounted on aluminum specimen stubs using conductive carbon adhesive tape and sputter-coated with a thin layer of gold using a sputter coater (Quorum Technologies Ltd., Laughton, East Sussex, United Kingdom). Fractographic evaluation was subsequently performed using a scanning electron microscope (JEOL JSM-IT200, JEOL Ltd., Tokyo, Japan) at magnifications ranging from 100× to 1,000×. Scanning electron microscopy (SEM) examination was performed to qualitatively evaluate the topographic characteristics of the fractured surfaces and identify the classical features associated with cyclic fatigue failure, including crack initiation zones, fatigue striations, microvoid coalescence, overload zones, and dimpled fracture morphology. The SEM observations were descriptive in nature and were not subjected to statistical analyses.

To ensure methodological standardization, the cyclic fatigue testing apparatus was calibrated before the initiation of the study by performing pilot runs using unused rotary instruments to verify alignment, rotational stability, canal dimensions, and reproducibility of fracture location. All the instruments were tested using the same artificial canal, endodontic motor, lubricant, environmental conditions, and testing sequence. Measurement reliability was enhanced by employing two independent investigators during each experiment: one responsible for operating the testing apparatus and recording the fracture time, and the second responsible for independently confirming the occurrence of fracture. Any discrepancy between the recorded observations was immediately resolved through consensus. As the primary outcome variables consisted of objective measurements generated under standardized laboratory conditions, no intra- or interexaminer reliability coefficients were required. Nevertheless, strict adherence to standardized protocols throughout the investigation ensured excellent experimental reproducibility. Similar fracture locations were observed in all groups, together with characteristic SEM fracture patterns, further confirming the consistency of the testing protocol.

The primary outcome measures included time to fracture (seconds) and NCF. Time to fracture represented the duration for which each instrument withstood continuous rotation before separation, whereas NCF provided a standardized measure of cyclic fatigue resistance by accounting for the differences in rotational speed among the tested file systems. Secondary qualitative assessment included SEM evaluation of the fracture morphology to characterize the mechanism of instrument failure.

Statistical analysis was performed using Statistical Package for the Social Sciences (version 25.0; IBM Corp., Armonk, NY). The normality of continuous variables was assessed using the Shapiro-Wilk test. Descriptive statistics were expressed as mean ± standard deviation (SD) together with 95% confidence intervals (CIs) for both the time to fracture and NCF. Intergroup comparisons were performed using a one-way ANOVA. Following a statistically significant ANOVA result, Tukey's honestly significant difference post hoc test was applied for pairwise multiple comparisons. A two-tailed p < 0.05 was considered statistically significant for all analyses.

## Results

Sixty NiTi rotary instruments were evaluated, with 20 instruments allocated to each study group. All specimens completed cyclic fatigue testing under standardized experimental conditions, and no instrument was excluded after allocation. Table 1 presents the descriptive statistics for the time to fracture and NCF. ProTaper Gold demonstrated the highest mean time to fracture (806.80 ± 58.65 seconds) and a high mean NCF (3,962.40 ± 297.80). Orodeka Plex exhibited the highest mean NCF (4,153.25 ± 421.74) with an intermediate time to fracture (514.65 ± 51.56 seconds), whereas GenEndo showed the lowest values for both time to fracture (406.80 ± 66.02 seconds) and NCF (2,619.20 ± 437.56).

**Table 1:** Descriptive analysis of time to fracture and NCF among the three rotary file systems Data are presented as mean ± SD with 95% CIs SD: standard deviation; CI: confidence interval; NCF: number of cycles to fracture

Groups	Time to fracture (seconds)		NCF	
	Mean ± SD	95% CI	Mean ± SD	95% CI
GenEndo	406.80 ± 66.02	375.90-437.70	2,619.20 ± 437.56	2,414.41-2,823.99
Orodeka Plex	514.65 ± 51.56	490.52-538.78	4,153.25 ± 421.74	3,955.87-4,350.63
ProTaper Gold	806.80 ± 58.65	779.35-834.25	3,962.40 ± 297.80	3,823.02-4,101.78

Table 2 summarizes the intergroup comparisons using one-way ANOVA. Statistically significant differences were observed among the three rotary file systems for both NCF and time to fracture (p < 0.001). The effect sizes were large for both outcome measures, indicating that the type of rotary file system had a substantial influence on cyclic fatigue resistance.

**Table 2:** Comparison of time to fracture and NCF (seconds) among the three rotary file systems using one-way analysis of variance Data were analyzed using one-way ANOVA. Effect size is expressed as eta squared (η²) ^*^p < 0.05 was considered statistically significant df: degrees of freedom; ANOVA: analysis of variance; NCF: number of cycles to fracture

Parameter	Source	Sum of squares	df	Mean square	F value	p value	Effect size
NCF	Groups	27,959,462.43	2	13,979,731.22	91.57	<0.001^*^	0.76
	Residual	8,702,255.75	57	152,671.15			
Time to fracture (seconds)	Groups	1,713,221.63	2	856,610.82	245.75	<0.001^*^	0.90

The pairwise comparisons obtained using Tukey's post hoc test are presented in Table 3. For NCF, significant differences were observed between GenEndo and Orodeka Plex (p < 0.001), and between GenEndo and ProTaper Gold (p < 0.001), whereas the difference between Orodeka Plex and ProTaper Gold was not statistically significant (p = 0.278). Regarding time to fracture, all pairwise comparisons demonstrated statistically significant differences (p < 0.001), with ProTaper Gold exhibiting the longest fracture time, followed by Orodeka Plex and GenEndo.

**Table 3:** Pairwise comparison of time to fracture (seconds) and the NCF among the three rotary file systems using Tukey's post hoc test Pairwise comparisons were performed using Tukey's honestly significant difference post hoc test following one-way analysis of variance. Data are presented as mean difference with corresponding 95% CIs ^*^p < 0.05 was considered statistically significant CI: confidence interval; NCF: number of cycles to fracture

Parameter	Paired group	Mean difference	t-value	p value	95% CI lower limit
NCF	GenEndo - Orodeka Plex	-1,534.05	-12.42	<0.001^*^	1,236.71-1,831.39
	GenEndo - ProTaper Gold	-1,343.20	-10.87	<0.001^*^	1,045.86-1,640.54
	Orodeka Plex - ProTaper Gold	190.85	1.54	0.278	-106.49 to 488.19
Time to fracture (seconds)	GenEndo - Orodeka Plex	107.85	5.78	<0.001^*^	62.92-152.78
	GenEndo - ProTaper Gold	400.00	21.42	<0.001^*^	355.07-444.93
	Orodeka Plex - ProTaper Gold	292.15	15.65	<0.001^*^	247.22-337.08

**Figure 1:**
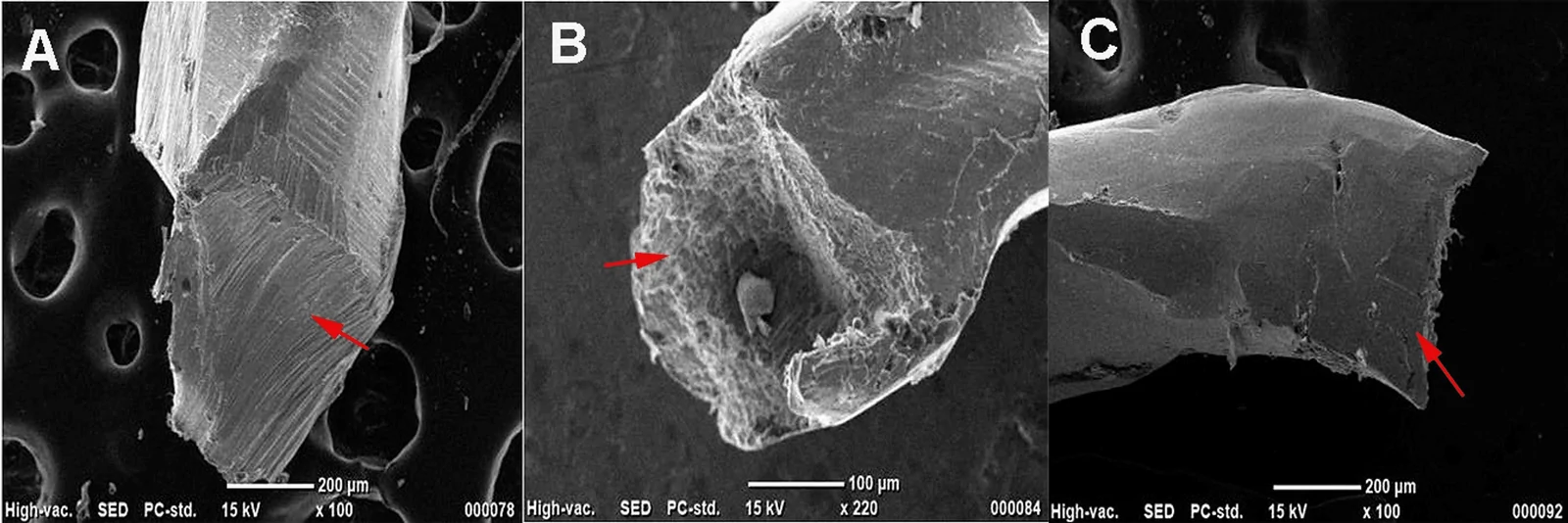
Original SEM images of the study sample of fractured NiTi rotary instruments following cyclic fatigue testing. (A) SEM micrograph at 100× magnification showing the fractured surface with characteristic fatigue features (red arrow) of GenEndo. (B) Higher magnification SEM image at 220× demonstrating the fractured surface and the crack initiation region (red arrow) of Orodeka Plex. (C) SEM micrograph at 100× magnification showing the fractured surface with evidence of the final overload fracture zone (red arrow) of ProTaper Gold SEM: scanning electron microscopy; NiTi: nickel-titanium

Representative SEM images of the fractured instrument are shown in Figure 1. SEM examination revealed the characteristic features of cyclic fatigue failure, including crack initiation areas, fatigue striations, and an overload fracture zone with a dimpled morphology. No evidence of plastic deformation or torsional failure was observed, confirming that the fracture occurred predominantly owing to cyclic fatigue under continuous rotational loading.

## Discussion

The present in vitro study compared the cyclic fatigue resistance of three contemporary heat-treated NiTi rotary file systems, GenEndo, Orodeka Plex, and ProTaper Gold, using a standardized simulated curved canal. Cyclic fatigue remains one of the principal causes of unexpected instrument separation during root canal preparation, particularly in severely curved canals, where files are subjected to repeated tensile and compressive stresses. Consequently, understanding the fatigue behavior of different rotary systems is essential for improving instrument selection and reducing procedural complications.

The findings demonstrated significant differences in cyclic fatigue resistance among the tested rotary file systems. ProTaper Gold exhibited the longest mean time to fracture, whereas Orodeka Plex demonstrated the highest mean NCF, with GenEndo showing the lowest performance for both parameters. One-way ANOVA confirmed statistically significant intergroup differences for both time to fracture and NCF, indicating that the mechanical performance of NiTi rotary instruments is substantially influenced by differences in alloy characteristics, heat treatment, cross-sectional geometry, and manufacturing processes [1].

The superior time-to-fracture performance observed for ProTaper Gold is likely attributable to its proprietary gold heat treatment and controlled memory (CM) metallurgy. Heat treatment modifies the transformation temperatures of the NiTi alloy, increasing its flexibility and reducing the internal stresses generated during repeated flexures in curved canals [10]. This improved flexibility delayed crack initiation and propagation, thereby extending the fatigue life of the instrument. Furthermore, the progressive taper design of ProTaper Gold distributes mechanical stresses more uniformly along the instrument, minimizing stress concentration at the point of maximum canal curvature. Similar findings have been reported by Uygun et al. [11], Ruiz-Sánchez et al. [12], and Ruiz-Sánchez et al. [13], all of which demonstrated significantly greater cyclic fatigue resistance for ProTaper Gold compared with conventional NiTi systems and several contemporary rotary instruments.

Keskin et al. [14] reported that thermally treated NiTi instruments exhibited significantly greater cyclic fatigue resistance than conventional NiTi systems under body-temperature conditions, attributing this improvement to enhanced flexibility and favorable phase transformation characteristics. These findings are in agreement with the present study, in which ProTaper Gold demonstrated the longest time to fracture among the tested instruments. The superior performance of ProTaper Gold may similarly be attributed to its proprietary gold heat treatment, which improves flexibility, reduces stress concentration during rotation in curved canals, and delays crack initiation and propagation, thereby enhancing resistance to cyclic fatigue.

Orodeka Plex demonstrated intermediate fatigue performance, with the highest calculated NCF but a shorter fracture time than ProTaper Gold. This apparent difference is primarily explained by the higher rotational speed recommended for Orodeka Plex (500 rpm) compared to ProTaper Gold (300 rpm) [15]. Because NCF incorporates rotational speed into its calculation, instruments operating at higher revolutions may accumulate more cycles before fracture, despite fracturing in a shorter period. The CM-wire alloy used in Orodeka Plex likely contributes to its favorable fatigue behavior by enhancing the flexibility and resistance to crack propagation [16]. Nevertheless, increased rotational speed may accelerate cyclic loading, thereby reducing the actual time required for fracture. These findings correspond with previous reports by Wang et al. [17], who observed moderate cyclic fatigue resistance for Orodeka Plex compared with other contemporary heat-treated rotary systems.

In this study, GenEndo demonstrated the lowest cyclic fatigue resistance among the three tested rotary file systems. This finding may be attributed to differences in alloy processing, heat treatment, cross-sectional geometry, core diameter, and manufacturing technology, all of which influence the stress distribution and crack propagation during continuous rotation in curved canals. Heat treatment modifies the phase transformation behavior of NiTi alloys by increasing the proportion of the martensitic phase at clinical temperatures, thereby enhancing flexibility and resistance to cyclic fatigue. Likewise, instruments with a smaller core diameter and optimized cross-sectional design exhibit lower stress concentration and greater elastic deformation, delaying crack initiation and propagation [6,12]. In contrast, variations in manufacturing processes, including machining quality and surface finishing, may introduce or reduce surface imperfections that act as stress concentrators, ultimately affecting the fatigue life of the instruments. Although published evidence regarding the mechanical properties of GenEndo instruments remains limited, Chhabra et al. [18] evaluated the shaping performance of GenEndo using cone-beam computed tomography and reported that it maintained canal anatomy with minimal canal transportation and satisfactory centering ability, comparable to ProTaper Gold, ProTaper Next (Dentsply Sirona Endodontics, Ballaigues, Switzerland), and NeoEndo Flex (Orikam Healthcare India Pvt. Ltd., Gurugram, India). These findings indicate that while GenEndo possesses favorable shaping characteristics, its cyclic fatigue resistance may differ from that of other contemporary heat-treated rotary systems because shaping ability and fatigue resistance are governed by different mechanical properties.

Consequently, an instrument capable of preserving the canal anatomy may not necessarily exhibit superior resistance to cyclic fatigue. SEM examination further supported the mechanical findings of the study. All the fractured specimens demonstrated classical fractographic features of cyclic fatigue, including crack initiation areas, fatigue striations, crack propagation zones, and overload fracture regions with a dimpled morphology. The absence of extensive plastic deformation or torsional abrasion confirmed that the instrument separation resulted predominantly from cyclic fatigue rather than torsional overload. These observations are consistent with numerous previous investigations evaluating NiTi rotary instruments and validating the reliability of the standardized testing protocol employed in the present study [3,4,7,10].

Although cyclic fatigue testing performed in artificial canals cannot completely reproduce the complex biomechanical environment encountered clinically, standardized laboratory models remain the most reliable method for comparing the intrinsic fatigue resistance of rotary instruments because they eliminate variability arising from differences in root canal anatomy, operator techniques, dentin hardness, and irrigation protocols. Therefore, the present results provide meaningful comparative information on the mechanical durability of the tested instruments.

Clinical implications

The findings of the present study have direct clinical relevance for endodontic practice. Rotary instruments with greater cyclic fatigue resistance are expected to exhibit improved durability during the instrumentation of severely curved canals and may reduce the likelihood of unexpected instrument separation. Understanding the fatigue characteristics of different NiTi systems enables clinicians to select instruments more appropriately according to canal complexity and anticipated mechanical stresses. The superior fatigue performance demonstrated by ProTaper Gold suggests that it may be a reliable option for preparing curved canals that require enhanced flexibility and resistance to cyclic fatigue.

Limitations

The present study had certain limitations. The investigation was performed using a static cyclic fatigue model, rather than a dynamic model, which may not completely reproduce the pecking motion employed during clinical instrumentation. Testing was conducted in a standardized stainless steel artificial canal that cannot fully replicate the anatomical complexity, dentinal properties, and temperature variations present in natural root canals. In addition, only one instrument size and canal curvature were evaluated. Future studies should investigate different instrument sizes, dynamic fatigue models, body temperature testing conditions, and combined cyclic and torsional fatigue assessments to provide a more comprehensive evaluation of the mechanical behavior of contemporary NiTi rotary systems.

## Conclusions

ProTaper Gold demonstrated the greatest resistance to cyclic fatigue by exhibiting the longest time to fracture, whereas Orodeka Plex showed the highest NCF owing to its higher rotational speed. GenEndo displayed comparatively lower fatigue resistance under standardized testing conditions. SEM confirmed the characteristic features of cyclic fatigue failure in all three systems. These findings highlight the significant influence of alloy metallurgy, heat treatment, rotational speed, and instrument design on the fatigue performance of NiTi rotary instruments, and provide useful evidence for informed instrument selection during endodontic treatment.
